# 

**Published:** 1803-08-01

**Authors:** 


### To CORRESPONDENTS.

Communications are received from Mr. G. N. Hill, Mr. A. Hunter, Mr. Aldus, Mr. Reid, and from the Continent.

